# Association between inflammatory bowel disease and bullous pemphigoid: a population-based case–control study

**DOI:** 10.1038/s41598-020-69475-0

**Published:** 2020-07-29

**Authors:** Yi-Ju Chen, Chao-Kuei Juan, Yun-Ting Chang, Chun-Ying Wu, Hsiu J. Ho, Hsiao-Ching Tseng

**Affiliations:** 1 Department of Dermatology, Taichung Veterans General Hospital, No. 1650, Sec. 4, Taiwan Blvd., Taichung, 407 Taiwan; 2 Faculty of Medicine and Institute of Clinical Medicine, School of Medicine, National Yang-Ming University, Taipei, Taiwan; 3 Department of Dermatology, Taipei Veterans General Hospital, Taipei, Taiwan; 4 Institute of Biomedical Informatics, Institute of Clinical Medicine, Institute of Public Health, National Yang-Ming University, Taipei, Taiwan; 5 Division of Translational Research and Center of Excellence for Cancer Research, Taipei Veterans General Hospital, No. 155, Sec. 2, Linong St., Beitou District, Taipei, 112 Taiwan; 6 Department of Public Health, China Medical University, Taichung, Taiwan; 7 National Institute of Cancer Research, National Health Research Institute, Miaoli, Taiwan; 8 Taiwan Microbiota Consortium, Taipei, Taiwan

**Keywords:** Diseases, Gastroenterology, Health care, Risk factors

## Abstract

The coexistence of inflammatory bowel disease (IBD) and bullous pemphigoid (BP) has been reported. No large-scale study to date has explored the relationship between these diseases. This population-based case-control study examined the association between IBD and BP by using a nationwide database. A total of 5,263 BP patients and 21,052 age- and gender-, hospital visit number-matched controls were identified in the National Health Insurance Research Database of Taiwan (1997–2013). Demographic characteristics and comorbidities including IBD were compared. Logistic regression was conducted to examine the predicting factors for BP. The mean age at diagnosis was 74.88 years and 54.3% of subjects were male. BP patients tended to have more cardiovascular risk factors, autoimmune and neurologic comorbidities, and hematologic cancers than matched controls. There were 20 cases of IBD (0.38%), mostly ulcerative colitis (N = 17, 0.32%) among BP patients, compared to 33 IBD cases (0.16%) among controls (p < 0.001). Ulcerative colitis was found to be significantly associated with BP [adjusted odds ratio (OR) 3.60, 95% confidence interval (CI) 1.91–6.77, p < 0.001] on multivariate analysis. Treatment for IBD was not associated with BP development. Information about diet, lifestyle, alcohol consumption, and smoking habit was not available. We concluded that UC is independently associated with BP.

## Introduction

Bullous pemphigoid (BP) is the most common autoimmune bullous disease. It mainly affects the elderly and is characterized by recurrent bullous lesions on trunk and limbs, with high morbidity and mortality. It is associated with autoantibodies against BPAG1 (also called BP 230/dystonin) and BPAG2 (BP180/type XVII collagen). The binding of autoantibodies to their targets activates inflammatory cascades that result in disruption of skin basement membrane integrity and subsequent subepidermal bullae formation^1^. The incidence is estimated to be 2.4–23 cases per million people in the general population but rises exponentially to 190–312 cases per million in the over 80 population^2^. A large body of evidence has suggested a strong association between BP and neurodegenerative diseases including dementia, cerebrovascular events, epilepsy, multiple sclerosis, and Parkinson's disease^2–6^. The molecular link between BP and neurodegenerative disorders has been hypothesized to be based on cross-reactivity with shared autoantibodies of BP 230 and BP180 expressed in neuronal and epithelial tissues^4^. Cardiovascular comorbidities have also been observed^3,6,7^. The potential mechanisms involve inhibited fibrinolysis and coagulation activation in BP patients and shared expression of BP230 in cardiac muscle and skin^8^. Advanced age at onset and frequent medical attention given to BP patients might also contribute to higher detection rates for comorbidities^4^.

Inflammatory bowel disease (IBD), which comprises both ulcerative colitis (UC) and Crohn's disease (CD), is a chronic relapsing inflammatory disease characterized by abdominal pain, diarrhea, bloody stool, weight loss, and fever^9,10^. IBD has been estimated to affect millions globally. Its incidence and prevalence have risen over more than a decade, especially among Asians^11,12^. The prevalence of CD in Taiwan has increased from 0.6/100,000 in 2001 to 3.9/100,000 in 2015. Likewise, the prevalence of UC in Taiwan has increased from 2.1/100,000 in 2001 to 12.8/100,000 in 2015^13^. IBD is hypothesized to occur as a result of an altered immune response to enteric bacteria in genetically susceptible subjects^14^. Cases of coexistent IBD and BP have rarely been reported^15–22^. It has been postulated that multiple antigens present in bowel epithelium, in which there is unregulated inflammation, result in activation of the immune system and subsequent recognition of autologous antigens^15^. However, there is a lack of large-scale studies on the link between IBD and BP.

In this study, we aimed to investigate the association between BP and IBD, as well as other cardiovascular risk factors and autoimmune diseases. Patients with BP and matched controls were included for comparison.

## Materials and methods

### Study design

We performed a nationwide case–control study by including patients with a diagnosis of BP and age-, gender, and hospital visit number-matched control subjects from Taiwan’s National Health Insurance Research Database (NHIRD) for the period 1997–2013. More than 99% of total population in Taiwan has been included in the National Health Insurance Database. (https://www.nhi.gov.tw) The NHIRD has been validated^23–25^ and utilized extensively in epidemiologic studies in Taiwan^26–28^. In this database, the diagnostic codes are in the format of the International Classification of Diseases, Revision 9, Clinical Modification (ICD-9-CM) with diagnoses made by board-certified physicians in the corresponding specialties. Personal information including body weight, height, family history, laboratory examination results, lifestyle, and habits such as smoking or alcohol use is not available from the NHIRD. This study has been approved by the institutional review board of Taipei Veterans General Hospital, Taipei, Taiwan (No. 2017-08-005CC).

### Case definition

All patients with a primary diagnosis of BP (ICD-9-CM code 694.5) for the first time between 1997 and 2013 were eligible for inclusion in this study. We included only those subjects who had received a major diagnosis of BP more than 3 times in an outpatient department or who had been admitted to a hospital for BP by dermatologist. Subjects with dubious basic data, such as conflicting gender or uncertain birth date, were not included. Patients ever receiving pathologic, direct immunofluorescence examinations or others were identified for additional sensitivity analyses.

The control group had no autoimmune bullous diseases, including pemphigus and BP, and, to reduce the selection bias, was randomly selected from the longitudinal health insurance database (LHID) 2000 and LHID 2010. LHID 2000 and LHID 2010 are subsets of NHIRD, which contain entire original claims data from 1,000,000 individuals randomly sampled from the Registry of NHIRD between 1996 and 2000, and 1,000,000 individuals randomly sampled in 2010, respectively. All sampled individuals were followed up from the date of entering NHI program to the end of 2013.

We matched each subject in the BP group with four control subjects without BP in the control group by age, gender and number of hospital visits. The date of the first BP diagnosis was defined as the index date.

Information regarding medications was retrieved from the pharmacy prescription database. Reliability of the retrieved information was independently verified by two statisticians.

### Covariate factors

Certain factors, such as age, gender, number of hospital visits, and comorbidities prior to the diagnosis of BP were considered potential confounders. Comorbidities included hypertension; diabetes mellitus; autoimmune diseases such as psoriasis, rheumatoid arthritis (RA), systemic lupus erythematosus (SLE), and Sjogren's syndrome (SS) diagnosed by dermatologists or rheumatologists; IBD diagnosed by gastroenterologists or colorectal surgeons; neurologic comorbidities such as stroke, dementia, epilepsy, and Parkinson's disease diagnosed by neurologists; and malignant diseases. All comorbid diseases were identified by at least 3 consecutive diagnoses in an outpatient department, by hospital admission, or from the Registry of Catastrophic Illness Patient Database, which is a separate subpart of NHIRD.

Insured patients who suffer from a major disease, such as malignancy, RA, SLE, Sjogren’s syndrome, stroke, multiple sclerosis, CD, or UC, can apply for a catastrophic illness certificate that grants exemption from all co-payments. Applications for catastrophic illness certificates must be validated by at least two specialists, based on careful examination of medical records, laboratory data, and imaging studies. Cytological or pathological reports or other evidence supporting the diagnosis are also required. Only those meeting the diagnostic criteria of a major disease are issued a catastrophic illness certificate. Both outpatient and inpatient claims by beneficiaries with a registered catastrophic illness are collected in the catastrophic illness profile and distributed as a package.

Patients with RA, SLE, SS, malignant diseases, stroke, multiple sclerosis, and IBD, including CD and UC, were identified from the Registry of Catastrophic Illness Patient Database.

Medications for IBD, such as 5-aminosalicylic acids (5-ASA), systemic corticosteroids, immunosuppressants, such as sulfasalazine, azathioprine, and cyclosporine; and biologics, such as anti-tumor necrosis factor α (TNF-α), were considered potential covariate factors for the development of BP. Long-term medication use was defined as use of a specific drug for more than one month after IBD diagnosis to the index date.

Further sensitivity analyses were conduced according to the status of further pathologic or other laboratory examinations in these BP patients.

### Statistical analysis

The demographic data of the study population were first analyzed. We compared the demographic factors and comorbid diseases between case and control groups by chi-square test. To assess the associations between covariate factors and BP, odds ratios (OR) and 95% confidence intervals (CI) were estimated using logistic regression method. All data management was performed with SAS 10.4 software (SAS Institute Inc., Cary, NC, USA).

## Results

### Demographic characteristics of study groups

The incidence of BP in Taiwan rises from 10 cases per million in 2000 to 28 cases per million in 2010. (Supplementary Table 1) Results of comparisons of demographic characteristics and associated coexistent major diseases between case and control groups are shown in Table 1. A total of 5,263 BP patients and 21,052 matched control subjects were enrolled. Patients in the BP group were more commonly associated with cardiovascular risk factors and neurologic comorbidities including stroke, Parkinson's disease, dementia, and epilepsy, with statistical significance. RA, psoriasis, rosacea, hematologic malignancies, and IBD were also more commonly seen in BP patients. BP patients with IBD were younger and had more malignancies than those without IBD. (Supplementary Table 2).

**Table 1:** Demographic characteristics of pemphigoid and control subjects.

	Pemphigoid (N = 5,263)	Control (N = 21,052)	p-value
Age, years, mean (SD)	74.88 (12.77)	74.86 (12.75)	0.916
Median (Q1–Q3)	78.02 (70.47–83.18)	78.02 (70.46–83.15)	0.887
**N (%) (years)**			
< 30	220 (1.05%)	55 (1.05%)	> 0.999
30–60	2,260 (10.74%)	565 (10.74%)	> 0.999
60–70	2,608 (12.39%)	652 (12.39%)	> 0.999
70–80	7,455 (35.41%)	1,855 (35.25%)	> 0.999
≥ 80	8,509 (40.42%)	2,136 (40.59%)	> 0.999
Male	2,859 (54.32%)	11,436 (54.32%)	–
N, hospital visits, mean (SD)	286.72 (251.15)	284.68 (220.41)	0.560
**Coexisting diseases**			
Hypertension	3,773 (71.69%)	13,771 (65.41%)	< 0.001
Diabetes mellitus	1855 (35.25%)	5,272 (25.04%)	< 0.001
ACS	1891 (35.93%)	7,921 (37.63%)	0.024
Psoriasis	121 (2.30%)	132 (0.63%)	< 0.001
Rosacea	16 (0.30%)	33 (0.16%)	0.042
RA	29 (0.55%)	59 (0.28%)	0.004
SLE	1 (0.02%)	10 (0.05%)	0.598
Sjogren’s syndrome	8 (0.15%)	19 (0.09%)	0.312
All cancers	327 (6.21%)	1,153 (5.48%)	0.041
Colon cancer	67 (1.27%)	283 (1.34%)	0.737
Hematologic cancer	17 (0.32%)	31 (0.15%)	0.013
Other cancers^a^	251 (4.77%)	863 (4.10%)	0.034
IBD	20 (0.38%)	33 (0.16%)	0.002
Ulcerative colitis	17 (0.32%)	25 (0.12%)	0.002
Crohn’s disease	3 (0.06%)	9 (0.04%)	0.942
Multiple sclerosis	1 (0.02%)	0 (0.00%)	0.453
Stroke	20 (0.38%)	4 (0.02%)	< 0.001
Dementia	862 (16.38%)	722 (3.43%)	< 0.001
Parkinson’s disease	574 (10.91%)	565 (2.68%)	< 0.001
Epilepsy	227 (4.31%)	143 (0.68%)	< 0.001

*ACS* acute coronary syndrome, *IBD* inflammatory bowel disease, *N* number, *Q* quartile, *RA* rheumatoid arthritis, *SD* standard deviation, *SLE* systemic lupus erythematosus.

^a^Others indicate all cancers excluding colon cancer and hematologic cancers.

### Univariate and multivariate analyses of risk factors for pemphigoid

Demographic characteristics and associated comorbid diseases listed in Table 1 were considered covariates for BP development. The mean time to development of BP for those with IBD was 4.38 years (Supplementary Table 3). Patients with IBD (adjusted OR 3.41, 95% CI 1.93–6.03, p < 0.001), especially UC (adjusted OR 3.60, 95% CI 1.91–6.77, p < 0.001), were independently associated with BP, after adjusting for the multiple covariate factors mentioned above (Table 2). CD is not found to be significantly associated with BP. Other independent risk factors for BP included stroke, Parkinson's disease, dementia, epilepsy, cardiovascular risk factors, RA, psoriasis, rosacea, and hematologic malignancies (Table 2).

**Table 2:** Multivariate analysis for predicting factors of pemphigoid.

	aOR^a^ (95% CI)	p value
Hypertension	1.26 (1.17–1.36)	< 0.001
Diabetes mellitus	1.62 (1.51–1.74)	< 0.001
ACS	0.81 (0.75–0.87)	< 0.001
Psoriasis	4.03 (3.10–5.24)	< 0.001
Rosacea	2.41 (1.29–4.51)	0.006
Rheumatoid arthritis	2.08 (1.30–3.33)	0.002
Hematologic cancer	2.68 (1.46–4.92)	0.002
IBD	3.41 (1.93–6.03)	< 0.001
Ulcerative colitis	3.60 (1.91–6.77)	< 0.001
Crohn’s disease	2.31 (0.62–8.58)	0.212
Stroke	18.20 (6.08–54.44)	< 0.001
Dementia	4.33 (3.87–4.85)	< 0.001
Parkinson’s disease	3.32 (2.90–3.80)	< 0.001
Epilepsy	4.82 (3.83–6.06)	< 0.001

*ACS* acute coronary syndrome, *CI* confidence interval, *IBD* inflammatory bowel disease, *aOR* adjusted odds ratio.

^a^Adjusted for factors including age, gender, number of hospital visits, cardiovascular risk factors, neurologic comorbidities, autoimmune diseases, and cancers listed in Table 1.

More than 90% of BP patients received the diagnosis from hospitals. A total of 82.8% BP diagnoses were made via pathologic or laboratory examinations including direct immunofluorescence or other circulating anti-basement membrane antibodies, in addition to the clinical diagnoses from dermatologists. And, 65.5% and 69.3% of BP patients received pathologic or laboratory examination within 3 months and 6 months, respectively, from the diagnosis. Further sensitivity analyses restricting BP patients with these examinations showed consistent results. (Supplementary table 4).

We next compared the long-term treatments for IBD between BP patients and controls (Table 3). No significant differences were observed between IBD patients with BP and without BP, in regards to long-term use of 5-ASA, corticosteroids, sulfasalazine, and azathioprine. No IBD cases were prescribed long-term cyclosporine or TNF-α antagonists.

**Table 3:** Association between IBD treatment and BP development.

Drugs for IBD^a^	BP with IBD (N = 20)	Controls with IBD (N = 33)	p value
5-ASA^a^	3 (15.0%)	7 (21.2%)	0.843
Salfasalazine	1 (5.0%)	3 (9.1%)	0.992
Meslamine	3 (15.0%)	5 (15.2%)	> 0.999
Balsalazine	1 (5.0%)	0 (0.0%)	–
Glucocorticosteroids	6 (30.0%)	5 (15.2%)	0.346
Azathiorpine	1 (5.0%)	0 (0.0%)	–
Antibiotics	14 (70.0%)	18 (54.5%)	0.409
Cyclosporine	0	0	–
TNF-α antagonists	0	0	–

*5-ASA* 5-aminosalicylic acid or mesalazine, *IBD* inflammatory bowel disease, *TNF-α* tumor necrosis factor alpha.

^a^Drug exposure for more than one month.

## Discussion

In this population-based case–control study, UC, but not CD, was found to be independently associated with BP. Long-term use of systemic 5-ASA, corticosteroids, and other immunosuppressants for the treatment of IBD may not affect BP development.

In contrast to the well-documented association between BP and neurologic diseases, only limited studies have reported the coexistence of BP and IBD^15–22^. One recent study utilizing nationally hospitalization dataset in the U.S. reported the association of autoimmune diseases in patients with pemphigus and pemphigoid^22^. UC was found to be associated with pemphigoid, especially in those under 50 years old^22^. However, we found no significant difference of age distribution between those with IBD and those without IBD in the BP group. (Supplementary Table 2) Another recent population-based study demonstrated an increased risk of autoimmune disease in IBD patients^10^. Over 70% of the autoimmune diseases in that study were of rheumatologic/connective or dermatologic origin. However, no specific diseases were mentioned. IBD patients of young age at onset (under 19 years) and of advanced age at onset (over 50 years) were mostly associated with autoimmune diseases^9,10^. Since our IBD cases developed BP in 4.87 years on average (Supplementary Table 2), they are assumed to be of advanced age at onset or to have delayed diagnosis of IBD. The coexistence of IBD and subepidermal bullous dermatoses, including BP, linear IgA dermatosis, and epidermolysis bullosa acquisita, has been reported^15^. It has been postulated that inflammation in IBD results in intestinal epithelial breakdown and exposure of basement membrane antigens to the immune system. The autoantigens in the gut may then be recognized in the skin, resulting in autoimmune bullous diseases^15^. This has been attributed to a cross-reactivity of antibodies, with BP180 and BP 230 expressed in gut and skin, based on human and murine studies^29,30^.

The importance of IL-12-type 1T helper (Th1) and IL-23-type 17T helper (Th17) pathways in the pathogenesis of UC and CD has been highlighted^31^, and emerging new treatments targeting these cytokine pathways for IBD are in development. It is hypothesized that the dysbiotic gut microbiome in IBD activates intestinal Th1 and Th17 inflammation. The mucosal injury then results in uptake of microbial antigen, toll-like receptor (TLR) ligand, and variable microorganisms that perpetuate immune responses^32^. Increased IL-17A, IL-17F, and IL-23A mRNA levels have been observed in inflamed UC and CD mucosa. Elevated toll-like receptor (TLR)-9 mRNA level has also been reported in inflamed CD ileal samples. Such aberrant inflammatory cytokine levels are related to clinical activities of UC and CD^33^. Likewise, dysregulation of IL-23-Th17 pathway has been proposed to be the key player in the pathogenesis of certain autoimmune diseases, such as ankylosing spondylitis, psoriasis, and psoriatic arthritis. One recent meta-analysis has demonstrated a bidirectional association between IBD and psoriasis, highlighting the shared inflammatory pathogenesis in both diseases^34^. Involvement of IL-17 signaling pathway in autoimmune bullous diseases, including pemphigus, BP, and dermatitis herpetiformis has been proposed^35–37^. Increased IL-17/IL-23 expression has been found in lesional skin and serum of BP patients at the time of diagnosis^35^. Persistent high serum IL-17/IL-23 levels in BP patients predict relapse after treatment^38^. Recently, one study using murine BP model demonstrated that anti-IL-17 drugs reduce inflammatory skin lesions and IL-17^−/−^ mice are protected from skin changes, such as erythema, erosion, and crust^36^. The common involvement of pro-inflammatory signaling in both diseases might reflect the nature of inflammation-driven or -facilitated bullous diseases after IBD.

Psoriasis and rosacea have been proposed to be risk factors for IBD in several large-scale studies^27,39^. After adjusting for these two confounding factors, the association between pemphigoid and IBD remained significant in the current study (Table 3). The link between psoriasis and BP has been reported previously^40^. However, the association between rosacea and BP has rarely been reported. A shared pathophysiological mechanism involving Th17 signaling pathway might partly explain the associations with BP. Furthermore, BP patients usually visit dermatologists more often than controls, and have more chance to be diagnosed with other skin diseases. We therefore matched subjects in both groups by the number of medical visits. Future cohort studies may be needed to clarify the issue.

Drug-induced BP should also be considered. Multiple drugs have been implicated in the pathogenesis of drug-induced BP, such as antibiotics, antidiuretics, antiarrhythmics/antihypertensives, neuroleptics, TNF-α antagonists, antidiabetics, antirheumatics, and salicylates, etc^18,41,42^. Association between 5-ASA (common drug used to treat IBD) and BP has been reported^42^. However, we did not find any associations for 5-ASA, corticosteroids, or immunosuppressants with BP development among our IBD cases (Table 3). The small number of IBD cases may have limited the impact of drug effect. Yet, this may imply that IBD itself, not the use of long-term 5-ASA, corticosteroids, or immunosuppressants, poses a risk for future BP development.

In contrast to the link between IBD and subepidermal bullous diseases, the association between IBD and pemphigus has rarely been reported^43^. We also conducted a case–control study to investigate the association between IBD and pemphigus using the same method. No significant differences in IBD cases were observed between pemphigus patients and matched controls (Supplementary Table 4).

There were several limitations to this study. First, due to the rarity of both BP and IBD in Taiwan, the association between these two diseases might have been underestimated. Moreover, the large age differences at onset of these two diseases might mean that only the association between elderly onset IBD and BP was identified. Due to the limited observation time of less than 20 years, the association of young age at onset of IBD with BP may not have been adequately observed. Second, misclassification bias may occur due to the identification of study subjects by diagnostic codes made by specialists only. Not all BP diagnoses made by dermatologists were confirmed by pathologic or immunofluorescence examinations. However, the results were consistent on sensitivity analyses after restricting patients who received examinations mentioned above. Third, we did not know the disease severity or phenotypes of pemphigoid due to the lack of detailed information in the database. Fourth, it is difficult to infer causation between a drug of interest and risk of outcomes based on an observational study, without a random assignment of treatments. Confounding by indication may exist. Finally, subjects may have differed in measurable and unmeasurable ways. We did not have access to personal information such as lifestyle, diet, smoking habit, alcohol use, or records of disease severity. We selected subjects with comparable characteristics for the two groups by matching age, gender, and number of hospital visits. Additional multivariate analyses after adjustment for multiple confounding factors were conducted to determine the independent risk factors for BP.

In conclusion, this nationwide case–control study provides evidence of an association between BP and IBD, as well as neurologic and cardiovascular diseases. The clinical relevance remains to be examined in future studies with more IBD patients. Underlying mechanisms involving both diseases may be worth further investigation.

## Supplementary information

Supplementary information

## Data Availability

The utilization of National Health Insurance Research Database was based on the approval of Taipei Veterans General Hospital IRB No. 2017-08-005CC.
