# Supplementary material for: Association between inflammatory bowel disease and bullous pemphigoid: a population-based case–control study
Source: Sci Rep. 2020 Jul 29;10:12727. doi: 10.1038/s41598-020-69475-0 (PMC7391771; doi:10.1038/s41598-020-69475-0)
Supplement: Supplementary file 1 — Supplementary information [file 41598_2020_69475_MOESM1_ESM.docx]

**Article type:** Original article

**Title:** Association between inflammatory bowel disease and bullous pemphigoid: a population-based case-control study

Yi-Ju Chen,^1,2*^ Chao-Kuei Juan,^1,2^ Yun-Ting Chang,^2,3^ Chun-Ying Wu, ^2,4-7*^Hsiu J. Ho,^4^ Hsiao-Ching Tseng^4^

^1^Department of Dermatology, Taichung Veterans General Hospital, Taichung;

^2^Faculty of Medicine and Institute of Clinical Medicine, National Yang-Ming University, Taipei;

^3^Department of Dermatology, Taipei Veterans General Hospital, Taipei;

^4^Institute of Biomedical Informatics, Institute of Public Health, National Yang-Ming University, Taipei;

^5^Division of Translational Research and Center of Excellence for Cancer Research, Taipei Veterans General Hospital, Taipei;

^6^Department of Public Health, China Medical University, Taichung;

^7^Nationa Institute of Cancer Research, National Health Research Institute, Miaoli;

Taiwan Microbiota Consortium;

All in Taiwan

Supplementary table 1. The incidence of bullous pemphigoid, 1997-2013 and the coverage rates of NHIRD in Taiwan

| Years | N^a^ | NHIRD coverage* (%) | Beneficiers in NHIRD* | Total population † | Incidence rate/  1,000,000 PY |
| --- | --- | --- | --- | --- | --- |
| 1997 | 102 | 94.248 | 20,492,317 | 21,742,815 | 4.691204888 |
| 1998 | 151 | 94.658 | 20,757,185 | 21,928,591 | 6.88598734 |
| 1999 | 221 | 95.462 | 21,089,859 | 22,092,387 | 10.00344598 |
| 2000 | 251 | 96.068 | 21,400,826 | 22,276,672 | 11.26739218 |
| 2001 | 268 | 96.643 | 21,653,555 | 22,405,568 | 11.96131247 |
| 2002 | 314 | 97.108 | 21,869,478 | 22,520,776 | 13.94268119 |
| 2003 | 294 | 97.256 | 21,984,415 | 22,604,550 | 13.00623105 |
| 2004 | 325 | 97.554 | 22,134,270 | 22,689,122 | 14.3240448 |
| 2005 | 380 | 97.998 | 22,314,647 | 22,770,383 | 16.68834468 |
| 2006 | 452 | 98.286 | 22,484,427 | 22,876,527 | 19.7582439 |
| 2007 | 465 | 99.323 | 22,803,048 | 22,958,360 | 20.25405996 |
| 2008 | 486 | 99.483 | 22,918,144 | 23,037,031 | 21.09646855 |
| 2009 | 550 | 99.593 | 23,025,773 | 23,119,772 | 23.78916193 |
| 2010 | 624 | 99.621 | 23,074,487 | 23,162,123 | 26.94053563 |
| 2011 | 660 | 99.886 | 23,198,664 | 23,224,912 | 28.41776107 |
| 2012 | 170 | 99.850 | 23,280,949 | 23,315,822 | 7.291186217 |
| 2013 | 162 | 100.382 | 23,462,863 | 23,373,517 | 6.930921008 |

Abbreviations: N, number; NHIRD, National health insurance research database; PY, person-years.

^a^ The number indicated newly diagnosed BP at the specific year, not limited to the cases included in our study.

*Official data from National Health Insurance Administration, Ministry of Health and Welfare, Taiwan. NHIRD coverage rate was calculated by beneficiers in NHIRD at the specific year/total population of Taiwan at the specific year. The beneficiers included foreigners working in Taiwan.

†Official data from Ministry of Interior, R.O.C. Taiwan

Supplementary table 2. Characteristics of BP patients with and without IBD

|  | BP without IBD | BP with IBD | p- value† | BP with UC | p value† |
| --- | --- | --- | --- | --- | --- |
| Number | 5,243 | 20 |  | 17 |  |
| Male | 2,847 (54.30%) | 12 (60.00%) | 0.775 | 11 (64.71%) | 0. 538 |
| Age, mean (SD) | 74.89 (12.78) | 71.75±11.38 | 0.272 | 73.33±11.41 | 0.614 |
| Age, median (Q1-Q3) | 78.03 (70.50-83.19) | 71.08 (67.49-79.87) | 0.109 | 73.82 (68.87-81.66) | 0.385 |
| N (%) |  |  |  |  |  |
| 0-30, y | 55 (1.05%) | 0 | - | 0 | - |
| 30-50y | 232 (4.42%) | 1 (5.00%) | 0.217 | 1 (5.88%) | 0.774 |
| 50-60 y | 330 (6.29%) | 2 (10.00%) | 0.217 | 1 (5.88%) | 0.774 |
| 60-70 y | 646 (12.32%) | 6 (30.00%) | 0.217 | 4 (23.53%) | 0.774 |
| 70-80 y | 1,849 (35.27%) | 6 (30.00%) | 0.217 | 6 (35.29%) | 0.774 |
| >80 y | 2,131 (40.64%) | 5 (25.00%) | 0.217 | 5 (29.41%) | 0.774 |
| Pathologic or other† | 4,332 (82.62%) | 18 (90.00%) | 0.568 | 16 (94.12%) | 0.353 |
| Hypertension | 3,762 (71.75%) | 11 (55.00%) | 0.158 | 10 (58.82%) | 0.362 |
| Diabetes mellitus | 1,851 (35.30%) | 4 (20.00%) | 0.232 | 4 (23.53%) | 0.447 |
| ACS | 1,880 (35.86%) | 11 (55.00%) | 0.122 | 10 (58.82%) | 0.086 |
| Psoriasis | 121 (2.31%) | 0 | - | 0 | - |
| Rosacea | 16 (0.31%) | 0 | - | 0 | - |
| RA | 29 (0.55%) | 0 | - | 0 | - |
| SLE | 1 (0.02%) | 0 | - | 0 | - |
| Sjogren syndrome | 8 (0.15%) | 0 | - | 0 | - |
| All cancer | 322 (6.14%) | 5 (25.00%) | 0.003 | 5 (29.41%) | <.001 |
| Colon cancer | 66 (1.26%) | 1 (5.00%) | 0.624 | 1 (5.88%) | 0.539 |
| Hema cancer | 17 (0.32%) | 0 | - | 0 | - |
| Others* | 247 (4.71%) | 4 (20.00%) | 0.007 | 4 (23.53%) | 0.002 |
| Multiple sclerosis | 1 (0.02%) | 0 | - | 0 | - |
| Stroke | 20 (0.38%) | 0 | - | 0 | - |
| Dementia | 860 (16.40%) | 2 (10.00%) | 0.639 | 2 (11.76%) | 0.851 |
| Parkinson’s | 572 (10.91%) | 2 (10.00%) | >.999 | 2 (11.76%) | >.999 |
| Epilepsy | 227 (4.33%) | 0 | - | 0 | - |

Abbreviations: ACS, acute coronary syndrome; BP, bullous pemphigoid; IBD, inflammatory bowel disease; N, number; Q, quartile; RA, rheumatoid arthritis; SD, standard deviation; SLE, systemic lupus erythematosus; y, years.

* Others indicate all cancers excluding colon cancer and hematologic cancers.

†p value, compared to BP without IBD.

Supplementary table 3. Average time to develop bullous pemphigoid in specific patient

|  | Time to index date*, pemphigoid, y | Time to index date*, control, y | p value |
| --- | --- | --- | --- |
| **Hematologic cancers, N** | 17 | 31 |  |
| Mean±SD | 4.33±2.79 | 5.85±4.37 | 0.204 |
| Median (Q1-Q3) | 3.64 (2.46-5.31) | 4.91 (2.31-8.35) | 0.314 |
| **IBD, all, N** | 20 | 33 |  |
| Mean±SD | 4.38±3.40 | 5.24±3.43 | 0.379 |
| Median (Q1-Q3) | 3.86 (1.08-7.44) | 4.81 (2.72-7.50) | 0.339 |
| **Ulcerative colitis, N** | 17 | 25 |  |
| Mean±SD | 4.87±3.41 | 5.03±3.54 | 0.886 |
| Median (Q1-Q3) | 4.05 (2.30-8.16) | 4.68 (2.49-7.50) | 0.820 |
| **Crohn’s disease, N** | 3 | 9 |  |
| Mean±SD | 1.62±1.86 | 5.95±2.99 | 0.043 |
| Median (Q1-Q3) | 0.64 (0.54-2.20) | 5.42 (3.76-6.69) | 0.064 |

Abbreviation: IBD, inflammatory bowel disease; N, number; Q, quartile; SD, standard deviation.

*Index date indicates the diagnosis date of bullous pemphigoid or the correspondent dates for controls. Time to the index date of diseases listed above indicates the duration from the time of diagnosis of these diseases to the diagnostic date of bullous pemphigoid.

Supplementary table 4. Sensitivity analysis restricting patients receiving pathologic or laboratory verification

| Test time to BP Diagnosis* | IBD, aOR† (95% CI) | p value | UC, aOR† (95% CI) | p value |
| --- | --- | --- | --- | --- |
| 3M | 3.49 (1.73-7.06) | <.001 | 6.10 (2.70-13.79) | <.001 |
| 6M | 3.84 (1.92-7.70) | <.001 | 4.92 (2.29-10.59) | <.001 |
| Ever | 3.87 (2.05-7.31) | <.001 | 4.79 (2.37-9.68) | <.001 |

Abbreviations: aOR, adjusted odds ratio; CI, confidence intervals; IBD, inflammatory bowel diseases; M, months; N (%), number of included BP patients in this study; UC, ulcerative colitis.

*Verification by pathologic or immunofluorence examination within specific period of time to the BP diagnosis.

†adjusted by demographic characteristics, comorbidities and other covariates listed in table 1.

Supplementary table 5. Demographic characteristics of pemphigus and matched controls

|  | Pemphigus (N=1,523) | Control (N=6,092) | p value |  |
| --- | --- | --- | --- | --- |
| Age, y, mean±SD | 52.68±16.59 | 52.67±16.58 | 0.993 | |
| Male | 718 (47.14%) | 2872 (47.14%) | >.999 |  |
| Hypertension | 369 (24.23%) | 1871 (30.71%) | <.001 |  |
| Diabetes mellitus | 176 (11.56%) | 830 (13.62%) | 0.037 |  |
| ACS | 176 (11.56%) | 927 (15.22%) | <.001 |  |
| Psoriasis | 71 (4.66%) | 31 (0.51%) | <.001 |  |
| Rosacea | 15 (0.98%) | 17 (0.28%) | <.001 |  |
| RA | 3 (0.20%) | 12 (0.20%) | >.999 |  |
| SLE | 4 (0.26%) | 2 (0.03%) | 0.019 |  |
| Sjogren syndrome | 6 (0.39%) | 8 (0.13%) | 0.071 |  |
| All cancer | 41 (2.69%) | 171 (2.81%) | 0.875 |  |
| Colon cancer | 8 (0.53%) | 25 (0.41%) | 0.695 |  |
| Hematologic cancer | 3 (0.20%) | 5 (0.08%) | 0.426 |  |
| Others* | 30 (1.97%) | 142 (2.33%) | 0.452 |  |
| Multiple sclerosis | 1 (0.07%) | 1 (0.02%) | 0.860 |  |
| Stroke | 0 (0.00%) | 0 (0.00%) | - |  |
| Dementia | 17 (1.12%) | 36 (0.59%) | 0.042 |  |
| Parkinson’s disease | 15 (0.98%) | 38 (0.62%) | 0.179 |  |
| Epilepsy | 12 (0.79%) | 31 (0.51%) | 0.268 |  |
| IBD | 2 (0.13%) | 3 (0.05%) | 0.576 |  |
| Ulcerative colitis | 1 (0.07%) | 3 (0.05%) | >.999 |  |
| Crohn’s disease | 1 (0.07%) | 0 (0.00%) | - |  |

Abbreviations: ACS, acute coronary syndrome; IBD, inflammatory bowel disease; N, number; Q, quartile; RA, rheumatoid arthritis; SD, standard deviation; SLE, systemic lupus erythematosus; y, years.

* Others indicate all cancers excluding colon cancer and hematologic cancers.
